# Small RNA sequencing and degradome analysis of developing fibers of short fiber mutants Ligon-lintles-1 (*Li*_*1*_) and −2 (*Li*_*2*_) revealed a role for miRNAs and their targets in cotton fiber elongation

**DOI:** 10.1186/s12864-016-2715-1

**Published:** 2016-05-17

**Authors:** Marina Naoumkina, Gregory N. Thyssen, David D. Fang, Doug J. Hinchliffe, Christopher B. Florane, Johnie N. Jenkins

**Affiliations:** Cotton Fiber Bioscience Research Unit, USDA-ARS, Southern Regional Research Center, 1100 Robert E. Lee Blvd, New Orleans, LA 70124 USA; Cotton Chemistry and Utilization Research Unit, USDA-ARS, Southern Regional Research Center, 1100 Robert E. Lee Blvd, New Orleans, LA 70124 USA; Genetics and Sustainable Agriculture Research Unit, USDA-ARS, 810 Highway 12 East, Mississippi State, MS 39762 USA

**Keywords:** Cotton, Fiber, Elongation, miRNA, Degradome, *Li*_*1*_ and *Li*_*2*_ mutations

## Abstract

**Background:**

The length of cotton fiber is an important agronomic trait that directly affects the quality of yarn and fabric. Understanding the molecular basis of fiber elongation would provide a means for improvement of fiber length. Ligon-lintless-1 (*Li*_*1*_) and −2 (*Li*_*2*_) are monogenic and dominant mutations that result in an extreme reduction in the length of lint fiber on mature seeds. In a near-isogenic state with wild type cotton these two short fiber mutants provide an effective model system to study the mechanisms of fiber elongation. Plant miRNAs regulate many aspects of growth and development. However, the mechanism underlying the miRNA-mediated regulation of fiber development is largely unknown.

**Results:**

Small RNA libraries constructed from developing fiber cells of the short fiber mutants *Li*_*1*_ and *Li*_*2*_ and their near-isogenic wild type lines were sequenced. We identified 24 conservative and 147 novel miRNA families with targets that were detected through degradome sequencing. The distribution of the target genes into functional categories revealed the largest set of genes were transcription factors. Expression profiles of 20 miRNAs were examined across a fiber developmental time course in wild type and short fiber mutations. We conducted correlation analysis between miRNA transcript abundance and the length of fiber for 11 diverse Upland cotton lines. The expression patterns of 4 miRNAs revealed significant negative correlation with fiber lengths of 11 cotton lines.

**Conclusions:**

Our results suggested that the mutations have changed the regulation of miRNAs expression during fiber development. Further investigations of differentially expressed miRNAs in the *Li*_*1*_ and *Li*_*2*_ mutants will contribute to better understanding of the regulatory mechanisms of cotton fiber development. Four miRNAs negatively correlated with fiber length are good candidates for further investigations of miRNA regulation of important genotype dependent fiber traits. Thus, our results will contribute to further studies on the role of miRNAs in cotton fiber development and will provide a tool for fiber improvement through molecular breeding.

**Electronic supplementary material:**

The online version of this article (doi:10.1186/s12864-016-2715-1) contains supplementary material, which is available to authorized users.

## Background

Cotton is a major source of natural fibers used in the textile industry. Cotton fibers are single-celled trichomes that emerge from the ovule epidermal cells. About 25–30 % of the seed epidermal cells differentiate into spinnable fibers [1]. Lint fibers of the economically important *Gossypium hirsutum* generally grow about 35 mm in length. Cotton fiber development consists of four distinct but overlapping stages, including fiber initiation, elongation, secondary cell wall (SCW) biosynthesis, and maturation [2]. Fiber elongation starts on the day of anthesis and continues for about 3 weeks before the cells switch to intensive SCW cellulose synthesis. During peak elongation fiber cells can increase in length at rates of 2 mm per day or more depending on environment and genotype [1–3]. The rate and duration of each developmental stage are important to the quality attributes of the mature fiber. Cell elongation is crucial for fiber length, whereas SCW is important for fiber fineness and strength. Understanding the molecular basis of fiber elongation would provide a means for cotton breeders and researchers to improve the fiber length while maintaining yield and other fiber characteristics.

Genetic mutants are useful tools for studying the molecular mechanisms of fiber development. Our laboratory uses two short fiber mutants, Ligon lintless-1(*Li*_*1*_) and Ligon lintless-2 (*Li*_*2*_) as a model system to study fiber elongation [4–10]. Both *Li*_*1*_ and *Li*_*2*_ are monogenic and dominant mutations, resulting in an extreme reduction in the length of lint fiber to approximately 6 mm on mature seeds [11, 12]. Both mutations are located in the D_T_ subgenome of *G. hirsutum*: the *Li*_*1*_ gene is on chromosome 22 [6, 13, 14], whereas the *Li*_*2*_ gene is on chromosome 18 [7, 14–16]. Cytological studies of cotton ovules did not reveal much difference between mutants and their near-isogenic WT lines during initiation and early elongation up to 3 days post anthesis (DPA) [7, 13]. In a fiber developmental study Kohel and co-authors observed that the elongation pattern is similar and restricted in both, *Li*_*1*_ and *Li*_*2*_ fibers [17]. However, unlike the normal morphological growth of the *Li*_*2*_ plants, the *Li*_*1*_ mutant exhibits pleiotropy in the form of severely stunted and deformed plants in both the homozygous dominant and heterozygous state [6, 11, 12]. The near-isogenic lines (NILs) of *Li*_*1*_ and *Li*_*2*_ with the elite Upland cotton variety DP5690 previously used in our research [6, 7] provide an excellent model system to study mechanism of fiber elongation.

Micro RNAs (miRNAs) are a class of non-coding endogenous small RNA that post transcriptionally regulate target genes expression [18]. Plant miRNAs range in size from 20 to 24 nucleotides. They negatively regulate gene expression by either mRNA degradation or translation inhibition [19–22]. miRNAs play important roles in most biological processes such as development, cell proliferation, stress response and metabolism [23–26]. Recently, identification and characterization of miRNA involved in fiber initiation and development have attracted much attention. For example, a recent study shows that miR828 and miR858 might regulate homeologous MYB2 (homologous to Arabidopsis *GL1*) gene functions in cotton fiber development [27]. Another study revealed that miR156/157 family plays an essential role in fiber elongation; suppressing expression of miR156/157 resulted in the reduction of mature fiber length [28]. However, currently the mechanism underlying the miRNA-mediated regulation of fiber development is largely unknown. Therefore, using short fiber mutants for identification and analysis of new miRNAs may provide a new insight in fiber development process.

In this work small RNA libraries from developing fiber cells of short fiber mutants and wild type were sequenced. *Gossypium hirsutum* TM-1 genome was used for miRNA structural prediction. We identified 24 conservative and 147 novel families whose targets were confirmed through degradome sequencing. Expression levels of 20 miRNAs families were extensively tested through the fiber development time course in wild type and mutant plants. Correlation analysis between expression levels of miRNAs with fiber length of 11 diverse cotton cultivar revealed 4 miRNAs that were significantly correlated with fiber length.

## Results

### Deep sequencing of small RNA libraries from developing fibers of short fiber mutants and wild type

**Fig. 1:**
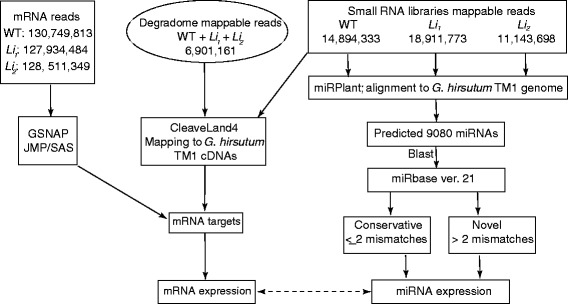
Flow chart of data processing to identify miRNAs and their targets in rapidly elongating cotton fiber cells transcriptome

We identified and tested expression level of miRNAs in rapidly elongating cotton fiber cells (at 8 DPA) of *Li*_*1*_, *Li*_*2*_ mutants and WT. The time point 8 DPA was selected because our earlier research revealed significant transcript and metabolite changes between the short fiber mutants and their WT NIL during this time of fiber development [7, 8, 10]. Three small RNA libraries were constructed and sequenced from developing fibers of *Li*_*1*_, *Li*_*2*_ and WT. The small RNA sequencing data were deposited into the National Center for Biotechnology Information (NCBI) with accession PRJNA307581. A total of 14,894,333, 18,911,773 and 11,143,698 mappable reads (about 80 % of total raw reads) were obtained from WT, *Li*_*1*_ and *Li*_*2*_ fiber cells, respectively. Figure 1 represents a flow chart of data processing to identify miRNAs and their targets. Mappable reads were run through miRPlant software for identification of plant miRNA from RNAseq data. *Gossypium hirsutum* TM-1 genome was used for mapping reads and prediction of hairpin structure of precursor’s miRNAs. A total of 9,080 miRNA sequences were predicted in three libraries (Additional file 1).

### Identification of conserved, previously reported and candidate miRNAs

**Fig. 2:**
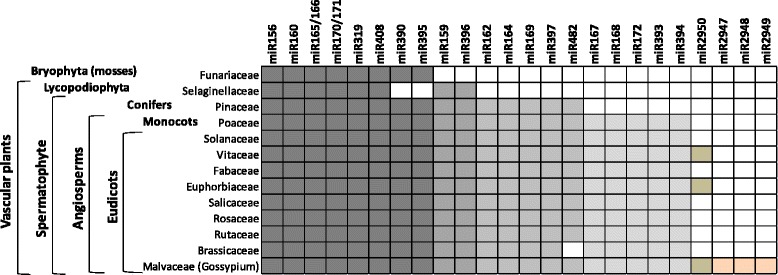
Deeply conserved and previously reported miRNA families detected in small RNA libraries from developing cotton fibers of WT, *Li*_*1*_ and *Li*_*2*_. miRNA families (columns) are conserved between plants families (rows) for plant species represented in miRBase release 21 [29]. Boxes are highlighted if a miRNA family was identified in at least one species for each plant families listed

We used the term “conserved” for miRNAs present in multiple species throughout at least one major ancient clade of land plants. To identify conserved and previously reported miRNAs, the 9,080 predicted miRNA sequences were BLAST searched against the miRNA sequences deposited in the miRBase release 21 [29]. The criteria of the BLAST search required no more than two mismatches with the sequences in miRBase. In total, 405 predicted miRNA sequences were identified as conserved or previously reported in miRBase release 21. The 405 redundant sequences were clustered into 24 known miRNA families (Fig. 2). Eight deeply conserved families were present across all land plants species; 2 families were present across vascular plants; 5 families were present across seed plants; 5 families were present across flowering plants; miR2950 was detected in eudicots and 3 families were previously reported in cotton. Six families, including miR159, miR165/166, miR167, miR168, miR390 and miR482, were the most abundant whose normalized expression levels were more than 30,000 reads per million (rpm) across 3 libraries (Table 1). After removing conserved and known miRNAs, the remaining sequences were clustered into 1726 candidate miRNA families. Additional file 2 provides information on loci of the candidate miRNA sequences in the TM-1 genome of and count of reads in each library.

**Table 1:** Target identification with degradome of highly expressed miRNAs

miRNA family	WT rpm	*Li*_*1*_ rpm	*Li*_*2*_ rpm	Degradome category	Degradome *p*-value	Degradome targets	Annotation
miR156/157	5379	4275	4799	4	0.016	Gh_D11G0401	SBP domain transcription factor
miR159	11904	10912	15397	4	0.005	Gh_A05G3434	MYB domain protein 33
miR160	445	389	630	0	0.001	Gh_D10G2093	auxin response factor 16
				4	0.018	Gh_A05G3576	auxin response factor 16
				4	0.021	Gh_A10G1836	auxin response factor 16
miR162	215	85	174	NA	NA	NA	
miR164	7071	9746	7036	4	0.010	Gh_D11G0347	NAC domain transcription factor
miR165/166	816686	871967	823901	NA	NA	NA	
miR167	11044	8262	13399	0	0.000	Gh_D07G1785	auxin response factor 8
				0	0.000	Gh_A12G0813	auxin response factor 6
				4	0.023	Gh_D12G0491	auxin response factor 8
				4	0.026	Gh_A12G0483	auxin response factor 8
				4	0.033	Gh_D05G0728	Glutathione S-transferase
miR168	32529	6047	13204	NA	NA	NA	
miR169	14	78	0	4	0.043	Gh_A11G0498	Predicted protein
miR171	143	93	174	0	0.000	Gh_A12G0855	GRAS family transcription factor
				0	0.000	Gh_D12G0935	GRAS family transcription factor
				4	0.028	Gh_A06G0358	GRAS family transcription factor
miR172	846	171	347	0	0.000	Gh_D08G0014	AP2 transcription factor
				4	0.01	Gh_D01G2112	AP2 transcription factor
miR319	186	295	239	4	0.018	Gh_D13G1576	TCP family transcription factor 4
				4	0.021	Gh_A01G0414	TCP family transcription factor 4
				4	0.026	Gh_A13G1272	TCP family transcription factor 4
miR390	66650	51554	43368	NA	NA	NA	
miR393	43	78	105	0	0.000	Gh_D07G2334	auxin signaling F-box 2
				0	0.000	Gh_A07G2125	auxin signaling F-box 2
				4	0.023	Gh_A11G0586	auxin signaling F-box 2
				2	0.043	Gh_A03G1585	Tubulin alpha-2 chain
miR394	14	54	63	0	0.000	Gh_A01G1280	F-box family protein
				2	0.043	Gh_A05G1551	Predicted protein
miR395	2223	2705	1672	NA	NA	NA	
miR396	7114	3552	6819	0	0.001	Gh_D05G0338	DENN (AEX-3) domain
				0	0.001	Gh_D12G2356	growth-regulating factor 8
				3	0.003	Gh_D04G1343	Predicted protein
miR397	273	218	158	NA	NA	NA	
miR408	86	178	105	2	0.027	Gh_A09G2056	Predicted protein
				4	0.046	Gh_A04G1079	Plantacyanin
miR482	12521	10205	13008	0	0.000	Gh_D13G2207	AP2/B3-like transcriptional factor
				0	0.000	Gh_A05G0923	Heavy metal transport
miR2947	1119	1617	1672	NA	NA	NA	
miR2948	731	404	282	NA	NA	NA	
miR2949	430	715	869	NA	NA	NA	
miR2950	344	194	152	3	0.008	Gh_A03G1623	Proline-rich cell wall protein
Novel_001	47	30	29	2	0.001	Gh_A01G0019	Plasma membrane intrinsic protein
Novel_002	42	20	38	2	0.05	Gh_A09G1276	Eukaryotic aspartyl protease
Novel_003	3218	8431	13506	2	0.044	Gh_D09G2263	Predicted protein
Novel_004	341	1314	6638	3	0.001	Gh_D13G2550	Glycine-rich RNA-binding protein
Novel_005	100	717	4205	0	0.020	Gh_D06G0870	glutathione S-transferase
Novel_006	16	335	1571	4	0.018	Gh_A10G0928	monodehydroascorbate reductase 4
Novel_007	89	278	374	3	0.05	Gh_D01G1810	3-ketoacyl-CoA synthase 6
Novel_008	47	74	125	4	0.041	Gh_D04G1574	Gibberellin-regulated family protein
Novel_009	341	1314	6638	4	0.048	Gh_A13G0294	Glycine-rich RNA-binding protein
Novel_010	94	788	3697	4	0.023	Gh_D01G0651	Predicted protein
Novel_011	105	228	603	3	0.05	Gh_D09G1279	Eukaryotic aspartyl protease
Novel_012	89	278	374	3	0.05	Gh_A01G1563	3-ketoacyl-CoA synthase 6
Novel_013	225	288	96	4	0.016	Gh_A07G0900	CAX interacting protein 4
				4	0.018	Gh_D07G0970	CAX interacting protein 4
Novel_014	26	87	345	2	0.05	Gh_A10G1097	Predicted protein
Novel_015	194	64	172	4	0.018	Gh_D02G0538	translationally controlled tumor protein
Novel_016	68	67	230	4	0.003	Gh_D12G0969	Nucleoside diphosphate kinase
Novel_017	16	80	268	0	0.05	Gh_A05G1124	Glucose-methanol-choline oxidoreductase
Novel_018	94	40	96	4	0.043	Gh_A10G1097	Predicted protein
Novel_019	21	87	105	4	0.041	Gh_A04G1428	Tonoplast monosaccharide transporter
Novel_020	42	54	86	4	0.021	Gh_D11G0401	SBP domain transcription factor
Novel_021	94	30	48	4	0.043	Gh_A10G1436	GTP1/OBG family protein
Novel_022	63	27	57	4	0.003	Gh_D05G0950	Predicted protein
Novel_023	5	13	96	2	0.013	Gh_A09G2056	Predicted protein
Novel_024	0	60	48	4	0.013	Gh_D05G1987	WPP domain protein 2
Novel_025	42	50	10	2	0.011	Gh_A11G2088	Predicted protein
Novel_026	0	23	77	2	0.045	Gh_A05G2976	Glyceraldehyde-3-phosphate dehydrogenase
				2	0.047	Gh_D04G0765	Glyceraldehyde-3-phosphate dehydrogenase

Degradome categories description: 0, more than 1 read equal to the maximum on the transcript when there is just 1 position at the maximum value; 1, more than 1 read equal to the maximum on the transcript when there is more than 1 position at maximum value; 2, more than 1 read above the average depth, but not the maximum on the transcript; 3, more than 1 read, but below or equal to the average depth of coverage on the transcript; 4, just one read at that position; rpm: reads per million.

### Target identification with degradome and selection of novel miRNAs

Given the high false-positive rate of computational predicted targets experimental confirmation of these targets is an important step. Degradome sequencing provides a high-throughput strategy for the global experimental identification of targets for miRNAs [30–32]. The degradome library was constructed from pooled RNA samples isolated from developing fiber cells at 8 DPA of WT, *Li*_*1*_ and *Li*_*2*_. The degradome reads were deposited into the NCBI with accession PRJNA307581. After removing low quality reads 6,901,161 mappable reads were obtained. We used the automated plant-compatible pipeline software CleaveLand4 to facilitate the interpretation of degradome data [33]. *G. hirsutum* TM-1 cDNA sequences were used for mapping degradome data (Fig. 1). Only miRNA targets with p-value ≤ 0.05 were used in further analyses. Candidate miRNA families whose cleaved targets were detected in degradome data were selected as novel miRNAs in this study. There were 147 novel miRNA families that met these requirements (Additional file 3).

**Fig. 3:**
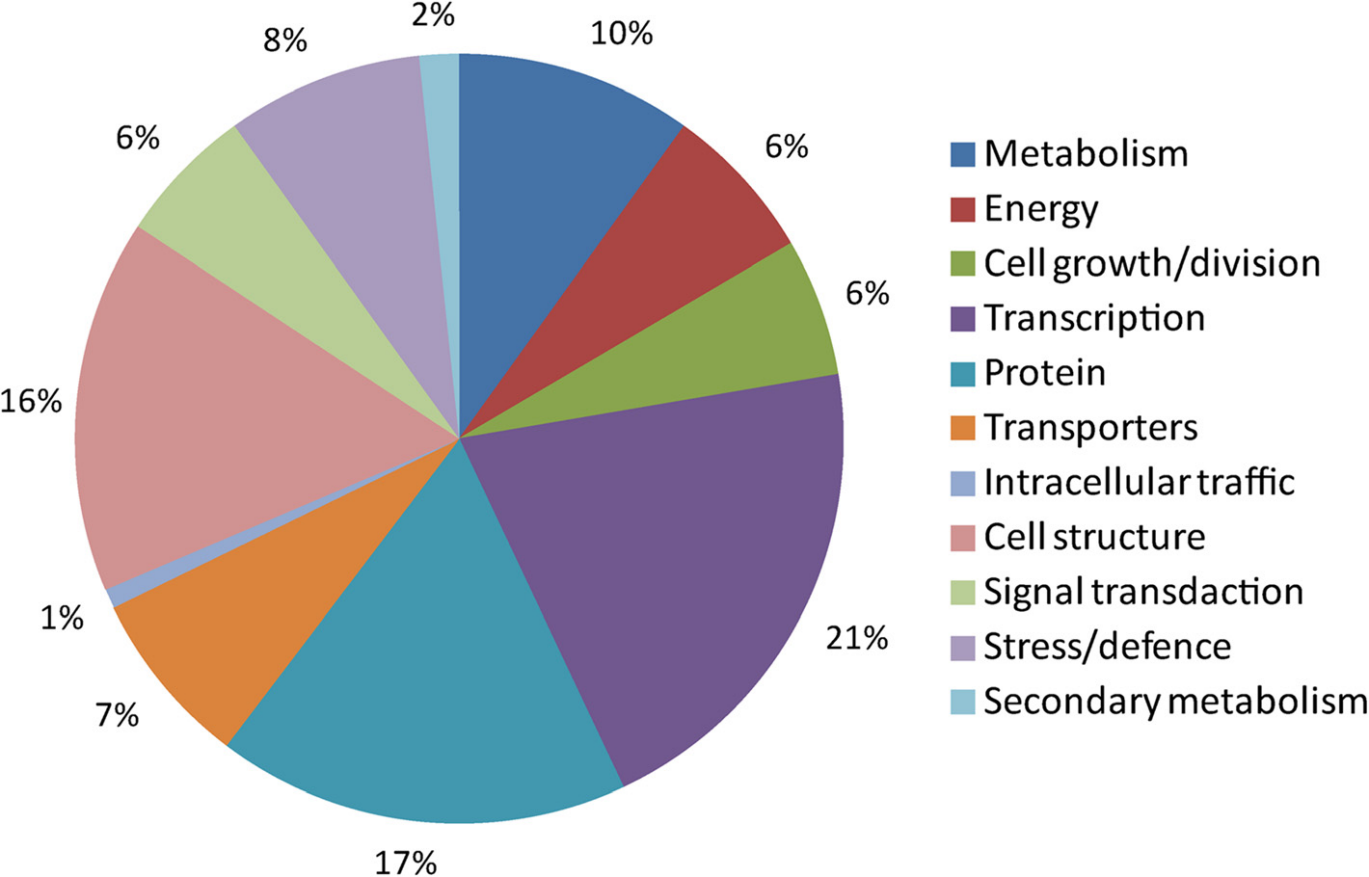
A pie chart showing functional categories of the target genes detected in degradome of rapidly elongating fibers of wild type and short fiber mutants

In total 157 non-redundant targets, including 36 targets of 24 known miRNAs and 121 targets of 147 novel miRNA families, were identified with degradome (Additional file 3). The target genes of known or predicted function were sorted into the 12 functional categories based on functional catalogues established for Arabidopsis [34]. The distribution of the target genes into functional categories is represented in Fig. 3. The largest set of genes (21 %) was assigned to the transcription category. The most abundant transcription factors (TFs) were auxin responsive, AP2, GRAS, and TCP (Additional file 3). Genes involved in protein biological processes and cell structure functional categories formed the second (17 %) and the third (16 %) largest groups (Fig. 3). Genes encoding structural constituent of cytoskeleton such as tubulin and actin were the most abundant (60 %) members of the cell structure functional category.

### Expression profiling of differentially expressed miRNAs in developing fibers of WT and short fiber mutants

**Fig. 4:**
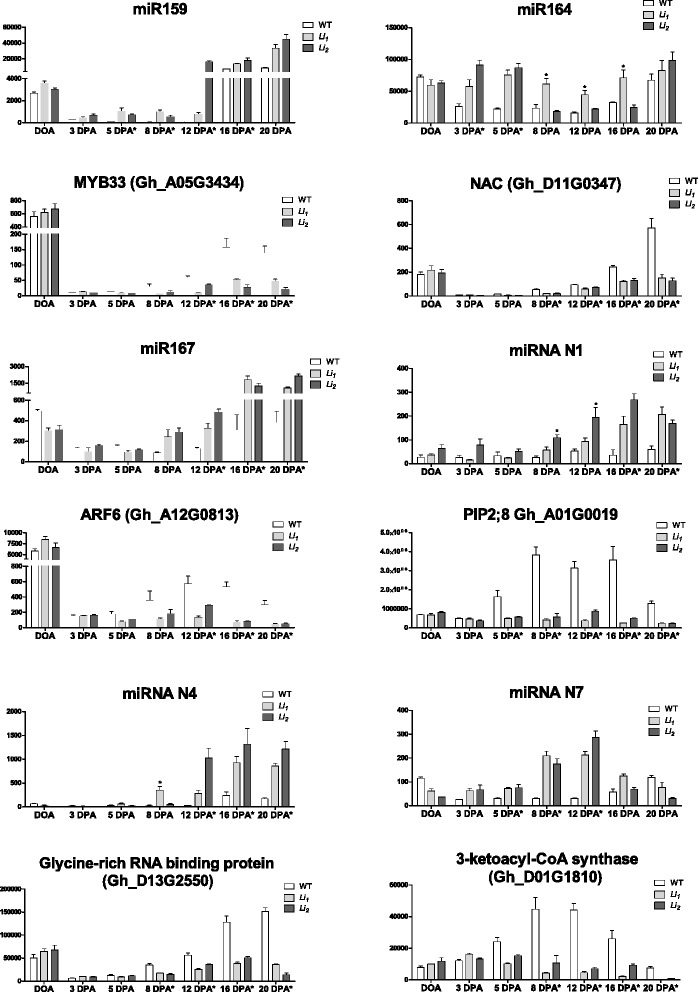
RT-qPCR expression analysis of miRNAs and their potential targets in developing cotton fiber cells of wild type and mutants. Each section represents expression profiles of miRNA and its target. The relative expression level is shown on the left *y*-axis of each graph. Asterisks indicate significant (p-value < 0.05) difference in gene expression level between mutant and wild type. Asterisks on x-axis represent significant difference in gene expression between wild type and both mutants, while asterisks on top of expression bars represent significant difference in gene expression between only one mutant line (bar with asterisk) and wild type. Error bars indicate standard deviation from 3 biological replicates

Expression levels of 11 conserved, 2 previously reported and 7 novel miRNA were tested by RT-qPCR in cotton fiber cells at 7 developmental time points (0, 3, 5, 8, 12, 16 and 20 DPA) of WT and short fiber mutants. The 20 miRNAs for RT-qPCR analysis were randomly selected from the Table 1. Expression profiles of miRNAs significantly differentially expressed between wild type and both mutants at multiple time points are shown in Fig. 4, whereas Additional file 4: Figures S1 and Additional file 5: Figure S2 provide RT-qPCR data for the rest of tested miRNAs. Overall, the majority of tested miRNAs exhibited higher expression levels during initiation (Day of anthesis, DOA) or transition to SCW deposition 16 – 20 DPA developmental stages. However, two identified miRNAs showed the elongation stage related pattern in short fiber mutants with transcript abundance decreasing at the beginning of the SCW stage from 16 – 20 DPA. The transcript abundance of Novel-7 (N7) miRNA was significantly increased (7.1-fold) in *Li*_*1*_ and (6.0 – 9.6-fold) in *Li*_*2*_ fiber cells at 8 – 12 DPA, consequently (Fig. 4). The transcript level of miR160 was significantly increased (3.2 – 4.9-fold) at 8 – 12 DPA, consequently, only in *Li*_*1*_ fiber cells (Additional file 4: Figure S1). The expression level of miR164 was significantly increased at 3 – 5 DPA in both mutants and at 8 – 16 DPA only in *Li*_*1*_ developing fibers. N1 and N4 miRNAs were up-regulated in mutants at the elongation (8 – 12 DPA) and the beginning of the SCW stages.

### Expression of target genes

To assess the influence of the miRNAs on their targets expression, we tested RNAseq expression of all target genes at peak of elongation (8 DPA), and evaluated by RT-qPCR the expression patterns of several selected target genes across different fiber developmental stages.

**Fig. 5:**
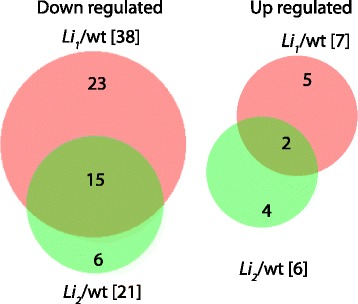
Venn diagrams of significantly down-regulated (left) and up-regulated (right) target genes in developing cotton fibers at 8 DPA of wild type and mutants. The total amount of down-regulated or up-regulated genes indicated in square brackets

To test the expression level of target genes we used RNAseq data from 9 libraries previously reported [10]. RNAseq libraries were constructed from developing fibers at 8 DPA of *Li*_*1*_, *Li*_*2*_ and WT in three biological replicates. Normalized expression data for 157 target genes, including least squares means, log2 ratios of comparisons mutants vs. wild type and *p*-values are provided in the Additional file 3. Of the 157 genes, 38 were significantly down regulated in *Li*_*1*_ and 21 in *Li*_*2*_ fiber cells and 15 were down regulated in both mutants (Fig. 5). Among the down-regulated genes in both mutant lines were plasma membrane intrinsic protein, 3-ketoacyl-CoA synthase, two eukaryotic aspartyl proteases, and tubulin. Among 23 down-regulated genes in *Li*_*1*_ were six tubulin genes, two 3-ketoacyl-CoA synthases, galactosyl transferase, and pectin lyase. Among 6 genes down-regulated specifically in *Li*_*2*_ were GRAS family TF and proline-rich cell wall protein. The number of genes significantly up-regulated in fibers of *Li1* and *Li2* were 7 and 6, respectively. Two genes of unknown function were up-regulated in fibers of both mutant lines (Fig. 5).

The transcript patterns of target genes of 6 miRNAs significantly up regulated in mutants fiber cells at multiple time points were evaluated by RT-qPCR (Fig. 4). TFs MYB33 (Gh_A05G3434), NAC (Gh_D11G0347) and ARF6 (Gh_A12G0813) were identified as targets of miR159, miR164 and miR167, respectively. Plasma membrane intrinsic protein 2;8 (PIP2;8, Gh_A01G0019) was identified as target of miRNA N1, whereas Glycine-rich RNA binding protein (Gh_D13G2550) and 3-ketoacyl-CoA synthase (Gh_D01G1810) were identified as targets of miRNAs N4 and N7, respectively. All tested targets revealed a negative relationship with corresponding miRNAs expression patterns (Fig. 4). The most interesting correlation pattern was observed between miRNA N7 and its target 3-ketoacyl-CoA synthase. The highest increase in transcript abundance of miRNA N7 was during peak of elongation (8 – 12 DPA), which corresponded to the lowest transcript abundance of 3-ketoacyl-CoA synthase during the same time points in mutants’ fiber cells.

### Correlation analysis between miRNA expression and fiber length

**Fig. 6:**
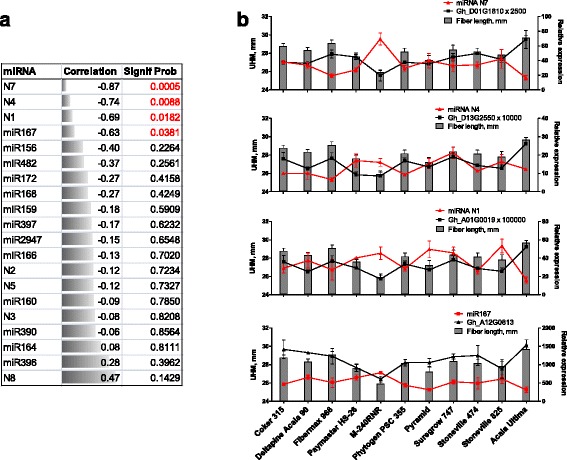
Negative correlation between miRNA expression level and fiber length of eleven diverse Upland cotton lines. The length of cotton fiber is shown on the left axis of each graph, whereas relative expression level of miRNAs (red line) and corresponding target (black line) is shown on the right axis of each graph. UHM, upper half mean length, the average length of the longer one half of the fibers sampled. Error bars for fiber length represent standard deviation between mean values from 3 years (2009–2011) of fiber measurements. Error bars for miRNAs or target genes expression represent standard deviation between 3 biological replicates

To investigate the affects of miRNAs expression levels on cotton fiber properties, we conducted correlation analyses between miRNAs transcript abundance and fiber lengths of 11 diverse Upland cotton lines. Fiber quality measurements were collected during 3 years (2009–2011) of growing plants in Starkville, MS field. For this study we used only measurements of the fiber length (upper half mean length, UHML). RNA for RT-qPCR expression analysis was collected from plants growing in Stoneville field in summer of 2015. Twenty above-described known and novel miRNAs identified in the study were used for correlation analyses. The correlation results, including Pearson coefficients and *p*-values, are represented in Fig. 6a. The expression patterns of 4 miRNAs, including N7, N4, N1 and miR167 revealed significant (*p*-value < 0.05) negative relationships with the fiber lengths of the 11 cotton lines. The transcript levels of targets of those 4 miRNAs were evaluated in the 11 cotton lines. As shown in Fig. 6b the transcript abundance profiles of targets revealed positive correlations with fiber length. The internal cleavage sites in the predicted targeted genes of N7, N4, N1 and miR167 miRNAs were confirmed by RNA ligase-mediated rapid amplification of 5’ and 3’ cDNA ends (RLM-RACE). The sequencing result of ten independent RACE fragments matched the site predicted by degradome analysis (Additional file 6: Figure S3).

## Discussion

The causative mutations of the cotton short fiber mutants *Li*_*1*_ and *Li*_*2*_ have yet to be identified. In this study we analyzed the small RNA libraries constructed from developing fibers of the two short fiber mutants and their WT NIL to examine possible regulation of genes by miRNAs during fiber elongation. We identified 24 conservative and 147 novel miRNA families in those small RNA libraries. Different phenotypic changes caused by the *Li*_*1*_ (dwarf deformed plants and short fiber phenotype) and the *Li*_*2*_ (short fiber phenotype only) mutations suggested that mutated loci are different types of genes. Expression profiles of a number of tested miRNAs were different in short fiber mutants than in WT during fiber development (Fig. 4, Additional file 4: Figures S1 and Additional file 5: Figure S2). These results suggested that the mutations changed the regulation of miRNAs expression during fiber development. Further investigations of differentially expressed miRNAs in the *Li*_*1*_ – *Li*_*2*_ mutants will contribute to better understanding of the regulatory mechanisms of cotton fiber development.

miRNAs negatively regulate gene expression by either mRNA degradation or translation inhibition. Previously, plant miRNA targets have been studied via computational prediction, which is based on sequence complementarity between miRNA and the target mRNA. Usually such predictions give high false-positive rates. Recently, a new method called degradome sequencing has been successfully established to screen for miRNA targets in plants. Degradome sequencing provides a high-throughput strategy for the global identification of small RNA-directed target cleavage by sequencing the 5’ ends of uncapped RNAs [30–33, 35]. In this study we have described only targets detected by degradome sequencing.

A large proportion of detected targets were TFs (21 %, Fig. 3), which was consistent with previous reports in cotton [36–38]. An essential role in fiber elongation has been illustrated for miRNA156/157 (targets SBP domain TF) in *Gossypium barbadense* [28]; however, our results demonstrated no significant difference in transcript abundance of miR156/157 in developing fibers of the short fiber mutants compared to WT fibers (Additional file 5: Figure S2). Therefore, miR156/157 most likely is not involved in the truncated fiber elongation caused by *Li*_*1*_ or *Li*_*2*_ mutations. In Arabidopsis, miR159 mediates cleavage of GAMYB-like genes that encode R2R3 MYB domain TFs that have been implicated in gibberellin signaling in anthers and germinating seeds [39]. Our results revealed miR159 significantly induced in short fiber mutants during elongation and SCW deposition stages (Fig. 4), suggesting involvement of miR159 in regulation of elongation. Previous studies in Arabidopsis demonstrated that the miR164 family guide the mRNA cleavage of five NAC TF genes that are required for boundary establishment and maintenance, lateral root emergence, formation of vegetative and floral organs, and age-dependent cell death [25, 40–42]. In this study, NAC domain TF (Gh_D11G0347) was among the predicted targets of miR164 (Table 1). Transcript level of miR164 was significantly up-regulated in the *Li*_*1*_ and *Li*_*2*_ fiber cells while its target NAC TF was down-regulated in mutant fibers (Fig. 4), suggesting the potential regulatory role of miR164 in fiber development.

There are very few studies that have performed a comparison of miRNAs expression profiles among different cotton genotypes with the exception of stress-related studies [43, 44]. So far only one report explored regulatory role of miRNAs in genotype-dependent traits in cotton [45]. In that study, the authors demonstrated that miRNAs have different expression patterns in different cotton varieties, which implicated their different phenotypic traits. In the current study we assessed whether miRNAs expression may be involved in the regulation of fiber length. Of the 20 tested miRNAs, 4 of them including miR167, N1, N4 and N7 had significant negative correlations with fiber lengths of 11 diverse Upland cotton lines (Fig. 6). The majority of fiber miRNA studies have focused on miRNA identification and expression analysis as well as target prediction and validation [37, 38, 46–49]. In addition to these observations the present study introduced correlation analysis with fiber lengths of commercially important cotton varieties that can be useful for plant molecular breeding. Our data demonstrated these 4 miRNA families were expressed more highly in the short fiber mutants at multiple time points of fiber development compared to the WT NILs (Fig. 4).

Auxin Response Factors (ARFs) were among the predicted targets of miR167. These proteins bind to the auxin response elements in the promoter regions of numerous early auxin-inducible genes [50, 51]. Exogenous auxin is required to promote fiber cell development from unfertilized ovules in culture [52]. Genetically engineered increase of auxin level in the epidermis of cotton ovules at the fiber initiation stage substantially increased the number of lint fibers and consequently fiber yield [53]. In Arabidopsis miR167 controls ARFs 6 and 8 expression patterns and affects the fertility of ovules and anthers [54]. Transgenic tomato plants over-expressing miR167 exhibited reductions in leaf size and internode length as well as shortened petals, stamens, and styles [55] that may cause infertility. In a comparative study of small RNA abundance between the wild-type and fuzz/lintless mutant, the expression of miR167 was significantly up-regulated in mutant fibers compared to the wild type [37]. A critical role of miR167 in cotton fiber elongation was suggested in another study exploring small RNA expression in developing fiber cells from 5 to 20 DPA [46]. Our data demonstrated that miR167 was significantly up-regulated in the *Li*_*1*_ and *Li*_*2*_ fiber cells while its target, ARF6 (Gh_A12G0813), was down-regulated in mutant fibers (Fig. 4). Also expression level of miR167 and its ARF6 target correlated with the fiber length of 11 cotton varieties (Fig. 6). This suggests that miR167 might be involved in regulation of fiber elongation.

Using correlation analysis we detected 3 more novel miRNAs which might regulate fiber length. Plasma membrane intrinsic protein PIP2;8 (Gh_A01G0019) was detected as a target of novel miRNA N1 by degradome analysis (Table 1). PIPs constitute a plasma-membrane specific subfamily of major intrinsic proteins or aquaporins which are associated with water transport and play important roles in fiber elongation. In our previous study, RNAseq analysis revealed that aquaporins were one of the most significantly over-represented gene families among down-regulated genes in *Li*_*1*_ and *Li*_*2*_ fibers [10]. The higher concentrations of inorganic ions detected in saps of fiber cells of *Li*_*1*_ – *Li*_*2*_ provided indirect evidence of reduced influx of water into fiber cells due to low expression of aquaporins and consequently a reduction in fiber cell elongation in mutants [10]. Our data have shown that the target of N1 miRNA Gh_A01G0019 is highly expressed during fiber elongation from 5 – 16 DPA in wild type fiber and exhibited greatly reduced expression in short fiber mutants (Fig. 4). The gene product of Gh_A01G0019 has 95 % amino acid sequence identity to a previously characterized PIP (PIP2;4), and it was shown that suppression of PIP2;4 expression by RNA interference markedly slowed down fiber elongation [56]. Therefore novel miRNA N1 represents a good candidate gene for further investigation of its role in regulation of PIP2;8 and fiber elongation.

The degradome detected target of novel miRNA N4 was glycine-rich RNA binding protein Gh_D13G2550. This gene has not been characterized in cotton and shows 82 % amino acid identity to Arabidopsis glycine-rich RNA binding protein 7 (AtGRP7). The AtGRP7 protein is regulated by circadian clock [57], involved in response to cold stress [58], and pathogen defence [59]. Transgenic Arabidopsis plants ectopically expressing AtGRP7 showed a dwarf phenotype due to distortions in gibberellin biosynthesis [60]. Our data have demonstrated that miRNA N4 transcript abundance was significantly higher in short fiber mutants during elongation – SCW deposition (8 – 20 DPA). The transcript abundance of its target was significantly reduced during the same period of time in mutants (Fig. 4). Therefore the miRNA N4 is another candidate for further investigations of its involvement in regulation of fiber elongation.

The novel miRNA N7 showed the most significant correlation probability with fiber length among tested miRNAs (Fig. 6). The degradome detected target of miRNA N7 was 3-ketoacyl-CoA synthase (KCS, Gh_D01G1810). KCS catalyses the initial condensation reaction during fatty acid elongation using malonyl-CoA and long-chain acyl-CoA as substrates [61]. Very long chain fatty acids significantly promoted cotton fiber cell elongation with several KCS genes highly up-regulated during cotton fiber development [62]. Our data have shown that the expression pattern of miRNA N7 revealed negative relationship with its target since the transcript abundance of KCS Gh_D01G1810 was highly increased during cotton fiber elongation (5–16 DPA) and significantly decreased in short fiber mutants (Fig. 4). This suggests that miRNA N7 might be involved in regulation of fiber elongation by targeting KCS.

## Conclusions

We identified 24 conservative and 147 novel miRNA families in small RNA libraries isolated from fiber cells of the cotton short fiber mutants *Li*_*1*_ and *Li*_*2*_ and their respective WT near-isolines. Fiber gene expression analysis of 20 selected miRNAs revealed differences in the expression profiles of short fiber mutants compared to WT during fiber development, which might reflect different transcript regulation in mutant lines comparing to WT fiber cells. Further investigations of these differentially expressed miRNAs will contribute to better understanding of the regulatory mechanisms of cotton fiber development. Of the 20 selected miRNAs, the expression patterns of 4 miRNA families showed significant correlations with fiber length of 11 eleven diverse Upland cotton lines. These miRNAs represent good candidates for further investigations of miRNA regulation of important genotype dependent fiber traits. The results of this study will contribute to further understanding of the role of miRNAs in cotton fiber development and will provide a tool for plant molecular breeding.

## Methods

### Plant materials

Two mutant lines *Li*_*1*_ and *Li*_*2*_ in a near-isogenic state with the WT Upland cotton line DP5690 were developed in a backcross program at Stoneville, MS as described before [6, 7]. A total of 150 *Li*_*1*_, 100 *Li*_*2*_, and 100 WT plants were grown in a field at the USDA-ARS Southern Regional Research Center, New Orleans, LA in the summer of 2013. First position flowers were tagged on DOA and bolls harvested at 0, 3, 5, 8, 12, 16, and 20 DPA. Bolls were randomly separated into 3 replicates with about 15–30 bolls per replicate. The ovules were carefully excised, immediately immersed in liquid nitrogen and stored at 80 °C.

Eleven diverse Upland cotton lines from across the United States were used for correlation analysis to compare the expression levels of miRNAs and the length of fiber. Lines were ‘Acala Ultima’, developed by California Planting Cotton Seed Distributors (Shafter, CA); ‘Tamcot Pyramid’, developed in the Multiple Adversity Resistant program by the Texas Agriculture Experiment Station, College Station. TX (Thaxton and El-Zik, 2004); ‘Coker 315’, developed by Coker Pedigreed Seed Co. (Hartsville, SC); ‘Stoneville 825’, developed by Stoneville Pedigreed Seed Co. (Stoneville, MS); ‘Fibermax 966’, developed by Bayer Crop Science (Lubbock, TX); M-240RNR, a root knot nematode resistant line developed by the ARS scientists Shepherd et al. [63]; ‘Paymaster HS-26’, a Texas High Plains cultivar developed by Paymaster Technologies, Inc. (Plainview, TX); ‘Deltapine Acala 90’, developed by Delta and Pine Land Co. (Scott, MS); ‘Sure-Grow 747’, developed by Sure-Grow Co. (Centre, AL) [64]; ‘Phytogen PSC 355’, developed by Mississippi Agriculture and Forestry Experiment Station (Mississippi State, MS) and licensed to Phytogen Seeds (Indianapolis, IN); and ‘Stoneville 474’, developed by Stoneville Pedigreed Seeds. Pedigrees for all except M-240RNR can be found in Bowman et al. [65].

For fiber quality measurements, seeds of the 11 cotton lines were planted as three replicates in a randomized complete block on the Plant Science Research Farm at Mississippi State, MS, in 2009, 2010 and 2011. Standard field practices were applied during the plant growing seasons. Twenty five healthy looking naturally opened bolls from the central part of a plant were hand harvested from each plant in 3 years. Boll samples were ginned on a 10-saw laboratory gin, and fiber properties were measured by Cotton Incorporated’s fiber measurement laboratory using a High Volume Instrument (HVI, USTER Technologies Inc., Charlotte, NC).

For RNA isolation from developing fibers seeds of 11 lines were planted in a field in Stoneville, MS, in 2015. Plants were in two- or four-row plots with ~70 plants per row. For the four-row plots, there were a total of three plots per cotton cultivar with each separate plot used as a biological replication. For the two-row plots there were a total of six plots with two plots used for each biological replication. First position flowers were tagged on the day of anthesis and bolls harvested at 10 DPA. Each biological replication consisted of about 8–15 bulked bolls. Once harvested, the bolls were placed immediately on ice in the field and transported to the laboratory. The bolls were dissected and the ovules with fibers attached were quickly frozen in liquid nitrogen and stored at −80 °C.

### RNA isolation and RT-qPCR

Total RNA was isolated from detached fibers [66] using the Sigma Spectrum Plant Total RNA Kit (Sigma-Aldrich, St. Louis, MO) with the optional on column DNase1 digestion according to the manufacturer’s protocol. The concentration of each RNA sample was determined using a NanoDrop 2000 spectrophotometer (NanoDrop Technologies Inc., Wilmington, DE). The RNA quality for each sample was determined by RNA integrity number (RIN) using an Agilent Bioanalyzer 2100 and the RNA 6000 n Kit Chip (Agilent Technologies Inc., Santa Clara, CA) with 250 ng of total RNA per sample. RNA from each of the above mentioned time-points was used for RT-qPCR analysis. A detailed description of reverse transcription and qPCR for quantification of mRNA transcripts was previously reported [7]. 18S rRNA was used as the endogenous reference gene for relative quantitation of the gene expression data.

A protocol published by Cirera and Busk [67] was used to quantify miRNA transcripts. Briefly, 100 ng of total RNA was incubated with 1 μL of 10x reaction buffer of *E. coli* poly(A) polymerase (New England BioLabs Inc., Ipswich, MA), 0.1 mM dNTP, 0.1 mM ATP, 1 μM universal RT primer (5’-CAGGTCCAGTTTTTTTTTTTTTTTVN), 1 U of *E. coli* poly(A) polymerase (New England BioLabs Inc.), and 100 U of M-MuLV reverse transcriptase (New England BioLabs Inc.) in 10 μL reaction mixture for 1 h at 42 °C. The reaction was inactivated by heating at 95 °C for 5 min and cDNA was diluted 50 times before being used in qPCR. Micro RNA-specific primers were designed with the miRprimer software [68]. The qPCR reactions were performed with iTaq™ SYBR® Green Supermix (Bio-Rad Laboratories Inc., Hercules, CA) in a Bio-Rad CFX96 real time PCR detection system. 5.8S rRNA was used as the internal reference gene for normalization of RT-qPCR data. The relative expression levels were calculated using the 2^-ΔΔCt^ [69]. Sequences of primers are listed in Additional file 7.

### Small RNA sequencing and processing

Small RNA libraries preparation and sequencing were conducted by LC Science (Houston, TX). RNA samples from three biological replicates extracted from developing fiber cells at 8 DPA were pooled together for preparation of WT, *Li*_*1*_ and *Li*_*2*_ small RNA libraries, respectively. The small RNA libraries were constructed using 1 μg of total RNA according to the TruSeq® Small RNA sample preparation guide (Illumina, San Diego, CA). The general process is as follows: first, the total RNA was ligated to RNA 3’ and RNA 5’ adapters. Second, reverse transcription followed by PCR was performed to create cDNA constructs based on the small RNAs ligated with 3’ and 5’ adapters. Third, small cDNA fractions that range from 22 nt to 30 nt in length were isolated by using 6 % denaturing polyacrylamide gel electrophoresis. Fourth, cDNA construct was purified, and the library was validated. The libraries were sequenced using Illumina Hiseq 2500 platform.

### Identification of conserved and novel miRNAs

Clean reads were run through miRPlant software for identification of plant miRNAs from small RNA sequencing data [70]. *Gossypium hirsutum* TM-1 genome [71] was used for mapping reads with software’s default parameters. Predicted by miRPlant miRNAs were BLASTed against the miRBase database (version 21, http://www.mirbase.org/) to identify conserved and previously reported miRNAs. Matched sequences with no more than two mismatches were considered as candidate conserved or previously reported miRNAs and were assigned to the corresponding miRBase family. Predicted miRNAs with more than 2 mismatches were considered as potential novel miRNAs because they lack sufficient similarity to assign to a miRNA family [72]. Statistical significance of differential expression of miRNAs in the sequencing data was established with the Audic & Claverie statistic using IDEG6 software [73, 74].

### Degradome library construction, sequencing, data analysis, and target identification

A degradome library was constructed from pooled RNA samples isolated from developing fiber cells at 8 DPA of WT, *Li*_*1*_ and *Li*_*2*_. The protocol is based on the method previously described by German et al. [35] and Addo-Quaye [30]. Briefly, poly(A)-enriched RNA was ligated to a 5’- RNA adapter with 3’ a EcoP15 I recognition site. Reverse transcription was performed to generate first-strand cDNA, followed by PCR amplification and EcoP15 I digestion. After digestion with EcoP15 I, a PAGE-gel was used to purify the EcoP15 I-cleaved fragments. The gel-purified products were ligated to a 3’-double-strand DNA adapter, followed by PAGE-gel purification to obtain the ligated products. PCR amplification was performed, and PAGE-gel was used for the third time to purify the corresponding gel bands containing the final products. Finally, the purified cDNA library was ready for deep sequencing. The library preparation and sequencing were conducted by LC Science (Houston, TX).

Single-end sequencing reads of 50 nucleotides were obtained using Illumina Hiseq 2500 platform. The adaptor sequences were trimmed from the raw reads and the reads shorter than 10 bases were excluded. Then these clean reads were mapped against the primary transcripts of *Gossypium hirsutum* TM-1 [71] using Bowtie [75]. A computational pipeline, CleaveLand [33], with its default parameters was used for the detection of cleaved miRNA targets from degradome data.

### 5’ RACE of miRNA cleavage

RLM-RACE was performed using the GeneRacer Kit (Invitrogen, Carlsbad, CA, USA) following the manufacturer’s instructions with minor modifications. Total RNA from developing fibers at 0, 3, 8 and 16 DPA was combined for mRNA isolation. Poly(A) mRNA was purified from total RNA using NucleoTrap mRNA Kit (Clontech, Mountain View, CA, USA) according to the manufacturer’s instructions. The GeneRacer RNA Oligo adapter was directly ligated to mRNA without calf intestinal phosphatase and tobacco acid pyrophosphatase treatment, which would have restricted analysis to full length mRNA. The GeneRacer Oligo dT primer was then used to synthesize first-strand cDNA. Two sets of reactions were performed: 1) with the GeneRacer 5’ Primer and gene-specific primers; and 2) with the GeneRacer 5’ Nested Primer and gene-specific nested primers (Additional file 7). After amplification, 5’RACE products were gel-purified and cloned into the pCR 4-TOPO vector, and approximately 10 independent clones were randomly chosen and sequenced.

### Availability of supporting data

The small RNA sequencing and degradome sequence data were deposited into the NCBI with accession PRJNA307581. RNAseq reads used for expression data are available at NCBI short reads depository with accession PRJNA273732.

## Additional files

Additional file 1: miRPlan predicted miRNAs from all mappable reads in 3 small RNA libraries. (TXT 7525 kb) Additional file 2: Novel miRNAs loci in TM-1 genome and reads count in each small RNA libraries. (TXT 142 kb) Additional file 3: Target identification with degradome conserved and novel miRNAs. RNAseq expression of target genes in elongating fiber cells at 8 DPA of *Li*_*1*_, *Li*_*2*_ and wild type. (XLSX 109 kb) Additional file 4: Figure S1. RT-qPCR expression analysis of highly expressed miRNAs in developing cotton fibers. The relative expression level is shown on the left *y*-axis of each graph. Asterisks indicate significant (p-value < 0.05) difference in gene expression level between mutant and wild type. Asterisks on x-axis represent significant difference in gene expression between wild type and both mutants, while asterisks on top of expression bars represent significant difference in gene expression between only one mutant line (bar with asterisk) and wild type. Error bars indicate standard deviation from 3 biological replicates. (PDF 38 kb) Additional file 5: Figure S2. RT-qPCR expression analysis of moderately expressed miRNAs in developing cotton fibers. The relative expression level is shown on the left *y*-axis of each graph. Asterisks indicate significant (p-value < 0.05) difference in gene expression level between mutant and wild type. Asterisks on x-axis represent significant difference in gene expression between wild type and both mutants, while asterisks on top of expression bars represent significant difference in gene expression between only one mutant line (bar with asterisk) and wild type. Error bars indicate standard deviation from 3 biological replicates. (PDF 44 kb) Additional file 6: Figure S3. Target gene validation by RLM-RACE. Gene map shows exons (ex) and miRNA target positions. The arrows indicate the cleavage sites and the number shows the frequency of the clones sequenced. (PDF 464 kb) Additional file 7: Primer’s sequences of miRNAs, their target genes and 5’ RACE gene specific primers. (XLSX 12 kb)

## Abbreviations

DOA: day of anthesis
DPA: days post-anthesis
KCS: 3-ketoacyl-CoA synthase
*Li*_*1*_: Ligon lintless-1
*Li*_*2*_: Ligon lintless-2
NILs: near-isogenic lines
RLM-RACE: RNA ligase-mediated rapid amplification of 5’ and 3’ cDNA ends
SCW: secondary cell wall
TF: transcription factor
WT: wild type
