# Supplementary figures and images for: Small RNA sequencing and degradome analysis of developing fibers of short fiber mutants Ligon-lintles-1 (Li1) and −2 (Li2) revealed a role for miRNAs and their targets in cotton fiber elongation
Source: BMC Genomics. 2016 May 17;17:360. doi: 10.1186/s12864-016-2715-1 (PMC4869191; doi:10.1186/s12864-016-2715-1)

**miR160**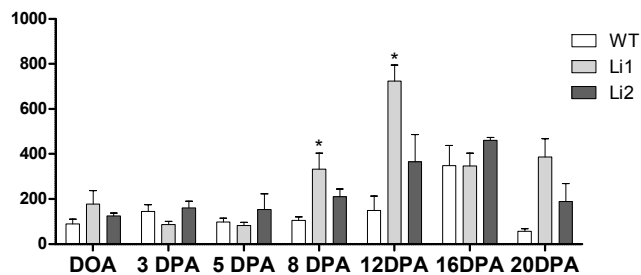**miR166**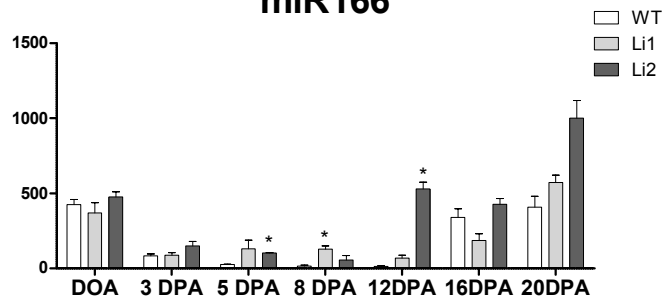**miR172**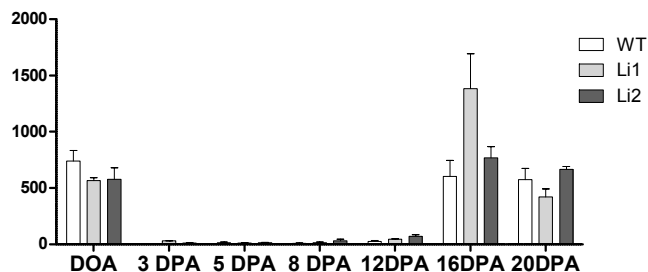**miR396**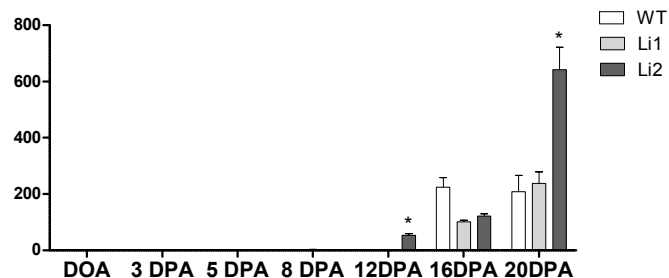**miR2947**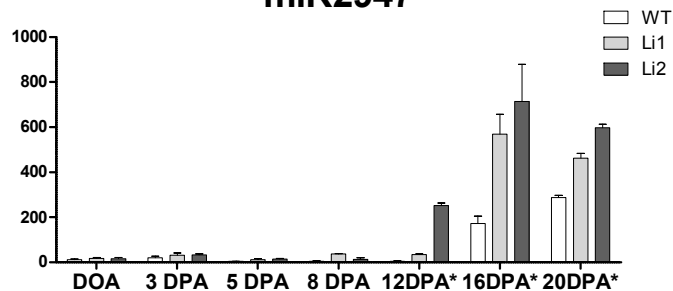**miRNA N3**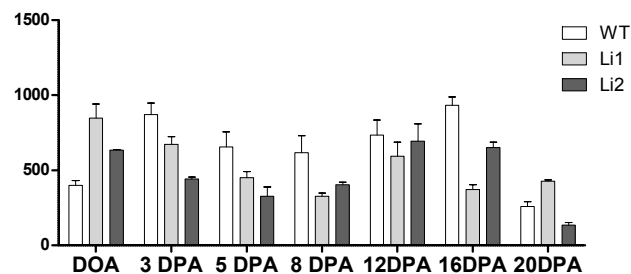

Supplement: Additional file 4: Figure S1. — RT-qPCR expression analysis of highly expressed miRNAs in developing cotton fibers. The relative expression level is shown on the left y-axis of each graph. Asterisks indicate significant (p-value < 0.05) difference in gene expression level between mutant and wild type. Asterisks on x-axis represent significant difference in gene expression between wild type and both mutants, while asterisks on top of expression bars represent significant difference in gene expression between only one mutant line (bar with asterisk) and wild type. Error bars indicate standard deviation from 3 biological replicates. (PDF 38 kb) [file 12864_2016_2715_MOESM4_ESM.pdf]

**miR156**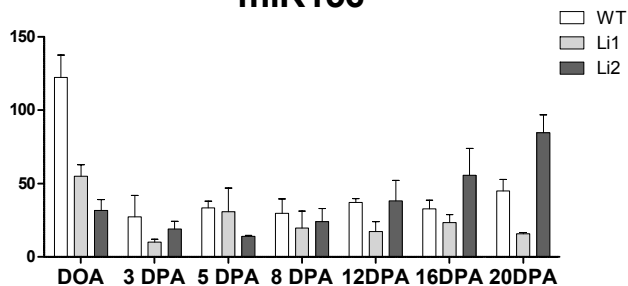**miR168**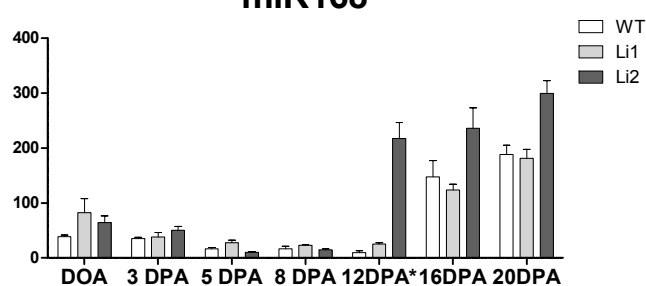**miR390**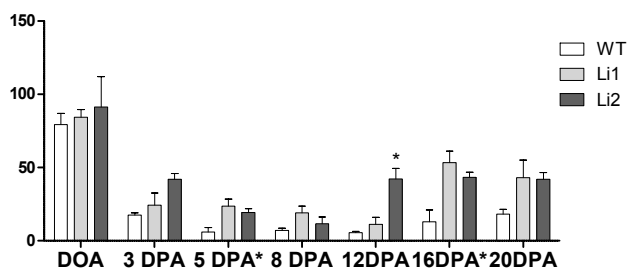**miR482**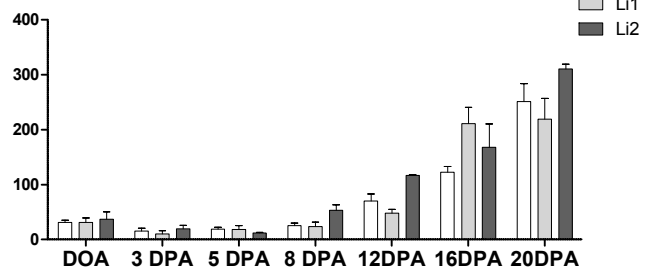**miR397**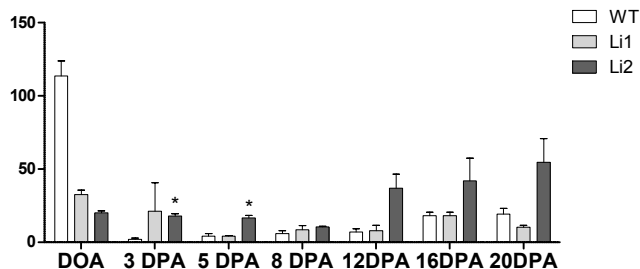**miRNA N2**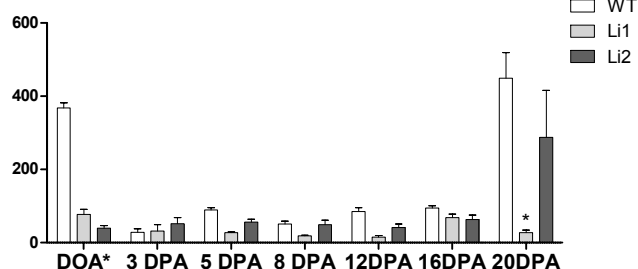**miRNA N5**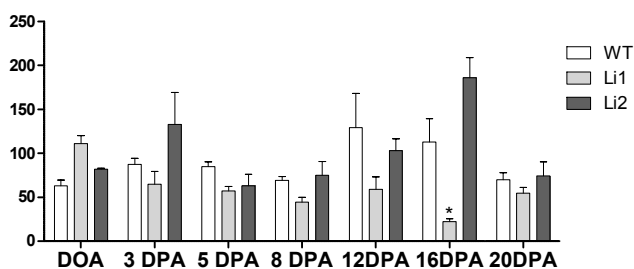**miRNA N8**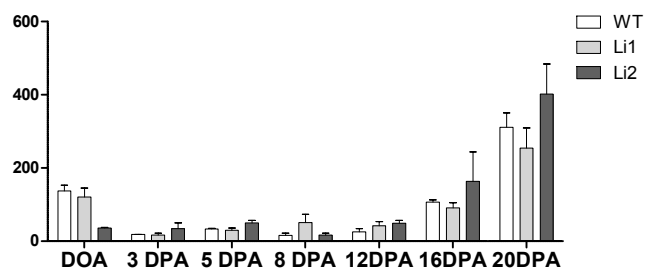

Supplement: Additional file 5: Figure S2. — RT-qPCR expression analysis of moderately expressed miRNAs in developing cotton fibers. The relative expression level is shown on the left y-axis of each graph. Asterisks indicate significant (p-value < 0.05) difference in gene expression level between mutant and wild type. Asterisks on x-axis represent significant difference in gene expression between wild type and both mutants, while asterisks on top of expression bars represent significant difference in gene expression between only one mutant line (bar with asterisk) and wild type. Error bars indicate standard deviation from 3 biological replicates. (PDF 44 kb) [file 12864_2016_2715_MOESM5_ESM.pdf]

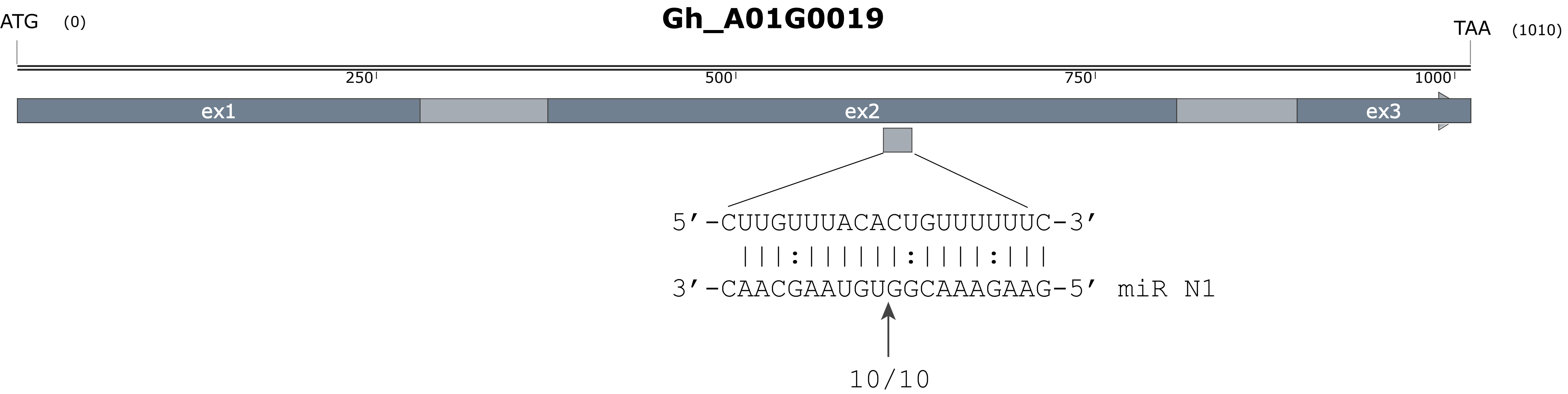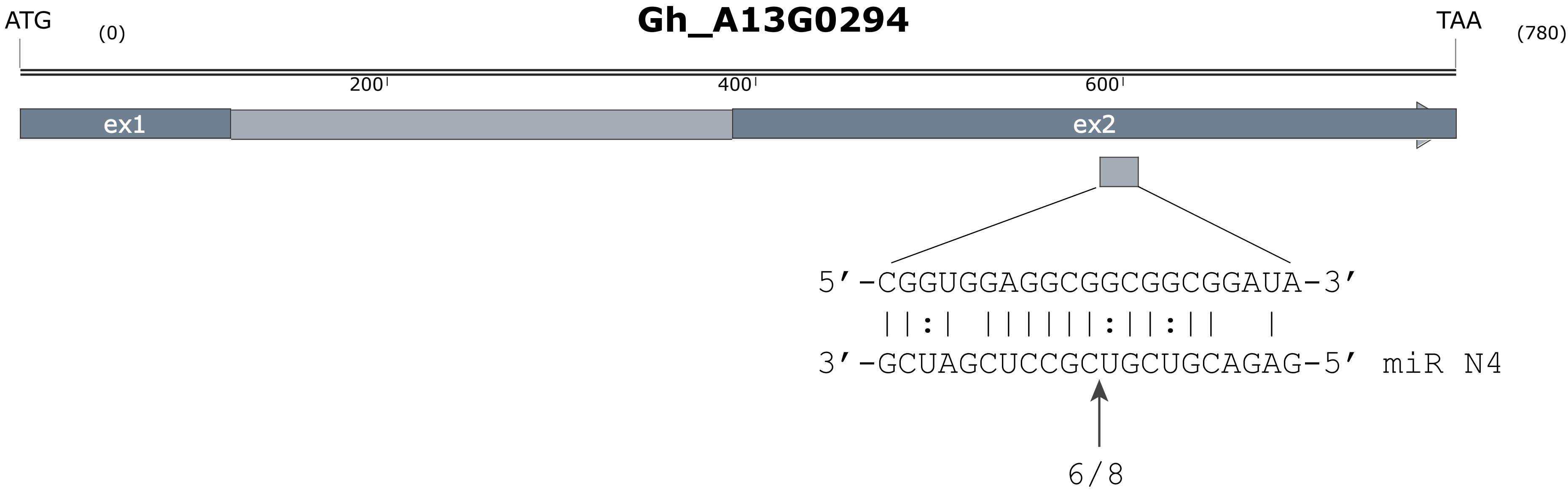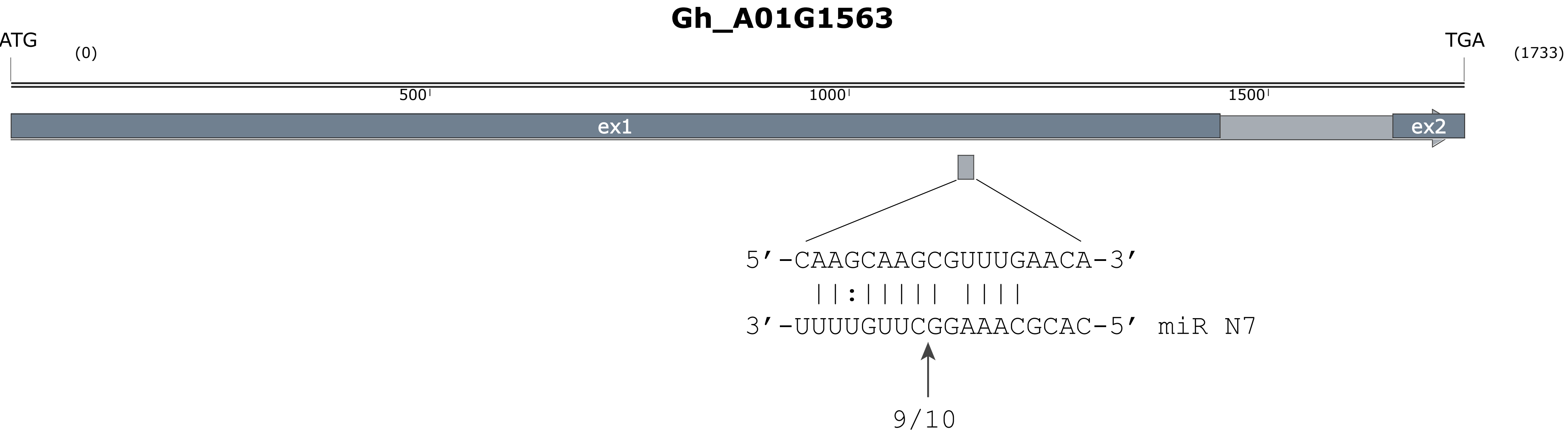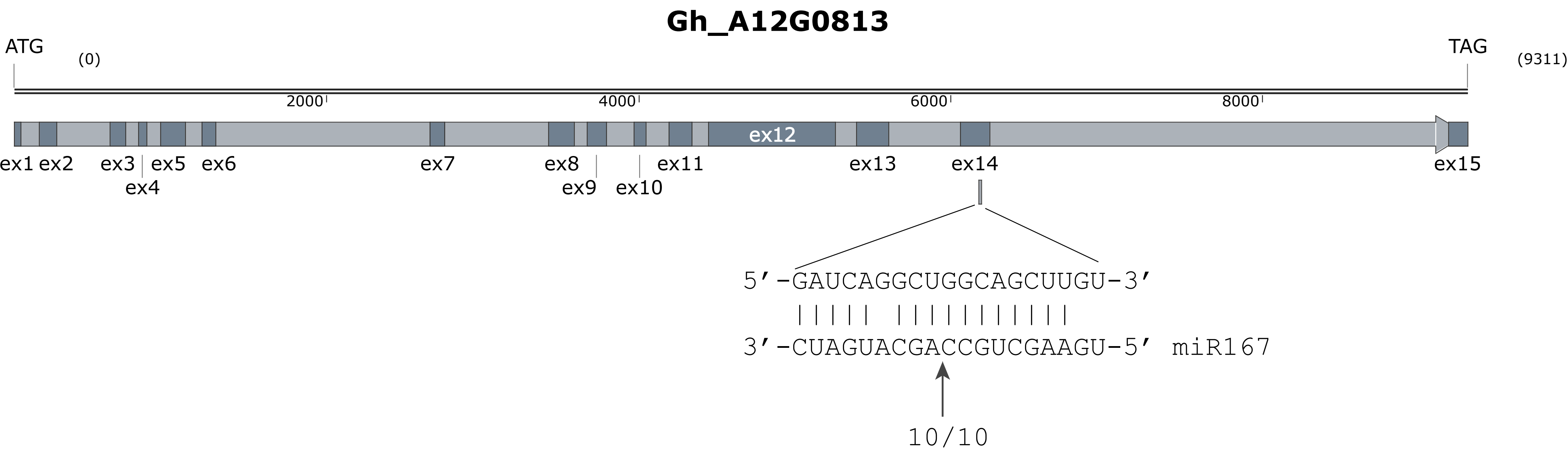

Supplement: Additional file 6: Figure S3. — Target gene validation by RLM-RACE. Gene map shows exons (ex) and miRNA target positions. The arrows indicate the cleavage sites and the number shows the frequency of the clones sequenced. (PDF 464 kb) [file 12864_2016_2715_MOESM6_ESM.pdf]
